# Modeling COVID-19 epidemics in an Excel spreadsheet to enable first-hand accurate predictions of the pandemic evolution in urban areas

**DOI:** 10.1038/s41598-021-83697-w

**Published:** 2021-02-22

**Authors:** Mario Moisés Alvarez, Everardo González-González, Grissel Trujillo-de Santiago

**Affiliations:** 1 Centro de Biotecnología-FEMSA, Tecnologico de Monterrey, 64849 Monterrey, NL Mexico; 2 Departamento de Bioingeniería, Escuela de Ingeniería y Ciencias, Tecnologico de Monterrey, 64849 Monterrey, NL Mexico; 3 Departamento de Ingeniería Mecatrónica y Eléctrica, Escuela de Ingeniería y Ciencias, Tecnologico de Monterrey, 64849 Monterrey, NL Mexico

**Keywords:** Applied mathematics, Scientific data, Infectious diseases, Epidemiology

## Abstract

COVID-19, the first pandemic of this decade and the second in less than 15 years, has harshly taught us that viral diseases do not recognize boundaries; however, they truly do discriminate between aggressive and mediocre containment responses. We present a simple epidemiological model that is amenable to implementation in Excel spreadsheets and sufficiently accurate to reproduce observed data on the evolution of the COVID-19 pandemics in different regions [i.e., New York City (NYC), South Korea, Mexico City]. We show that the model can be adapted to closely follow the evolution of COVID-19 in any large city by simply adjusting parameters related to demographic conditions and aggressiveness of the response from a society/government to epidemics. Moreover, we show that this simple epidemiological simulator can be used to assess the efficacy of the response of a government/society to an outbreak. The simplicity and accuracy of this model will greatly contribute to democratizing the availability of knowledge in societies regarding the extent of an epidemic event and the efficacy of a governmental response.

## Introduction

A SARS-CoV-2 (COVID-19) pandemic was declared by the World Health Organization in March 2020. More than 60,000,000 positive cases of COVID-19 infection had been declared worldwide at that point, mainly in China, Italy, Iran, Spain, and other European countries. By the end of 2020, one year after its emergence, the official cumulative number of infected worldwide ascended to more than 80 million with a toll of death higher than 1.75 million and a strong presence in Las Americas, mainly in the USA^1^, Europe, and India^2^. The socio-economic effects of COVID-19 have been and will be also remarkable^3,4^, and have to be yet fully quantified. COVID-19, the first pandemic of this decade and the second in less than 15 years, has harshly taught us that viral diseases do not recognize boundaries; however, they truly do discriminate between aggressive and mediocre containment responses.

Indeed, we have been able to observe exemplary responses from some Asian countries (i.e., China^5^, South Korea^6^, and Singapore^7^), some highly aggressive responses in Europe (i.e., Germany and Switzerland^8^), and several delayed or not so effective responses from other regions (i.e., USA, England, Italy and Spain)^9,10^. At this point, some territories in Latin America (i.e., México) are just experiencing a second “exponential phase” of the COVID-19 pandemic at home and do not appear having yet implemented proper containment measures as rapidly as needed.

The gap between developed and developing countries may explain some of the differences in the scale of the responses that we are observing^3^. Countries that are better equipped than others in terms of high-end scientific development, diagnostics technology, and health care infrastructure may respond more efficaciously to a pandemic scenario. However, other tools, such as mathematical modeling, are much more widely available and may be of extraordinary value when managing epidemic events such as the COVID-19 pandemics. To date, many papers have reported the use of mathematical models and simulators to evaluate the progression of COVID-19 in local or more global settings^11–14^. Predictions on the possible evolution of COVID-19 based on mathematical modeling could therefore represent important tools for designing and/or evaluating countermeasures^13,15–17^.

Historically, the use of models based on the definition of distinct and interacting compartments of susceptible, infected, and recovered individuals (SIR models) has been the preferred modeling strategy^18^. Variations of the original SIR model have been adapted to include other subpopulations, such as asymptomatic^2^ and exposed individuals^19^. These adapted models (i.e., SEIR models) have been remarkably useful for describing epidemic events and have contributed enormously to our understanding of epidemic progression^19^, COVID-19 included^20^. However, SIR-related models exhibit some limitations in the context of COVID-19 modelling^21^. For example, the progression of COVID-19 is eminently influenced by demographic factors^3,10^, whereas SIR-related models are not intrinsically demographic-based. In addition, SIR-related models do not explicitly account for the active infective role of asymptomatic individuals. This may lead to relevant inaccuracies, for example missing the occurrence of the epidemic plateau that has been frequently observed during COVID-19 progression in different regional settings^21^.

Mathematical modeling may (and probably should) become a much more available tool in the case of public health emergencies—one ideally widely available to practically any citizen in any of our societies. One decade ago, during the influenza pandemics, mathematical modeling of epidemic events was the realm of privileged epidemiologists who had (a) a fast computer, (b) programing experience, and (c) and access to epidemiological data. Today, these three ingredients are reduced to a convectional laptop, very basic differential equation-solving skills, and access to a website with reliable online statistical information on epidemics. The availability of a simple model may be highly enabling for local governments, physicians, civil organizations, and citizens as they struggle in their endeavor to accurately forecast the progression of an epidemic and formulate a plan of action. Friendly and widely available mathematical modeling will enable rational planning (i.e., prediction of hospital bed occupancy, design of testing campaigns, and reinforcement/redirection of social distancing strategies). Moreover, the use of simple/user-friendly models to evaluate in (practically) real time the effectiveness of containment strategies or programs may be a powerful tool for analyzing and facing epidemic events^11,17^. In addition, monitoring actual data, while comparing them with model predictions, enables real-time assessment of the effectiveness of the containment measures. In turn, this empowers officials, scientists, health care providers, and citizens.

The main purpose of this contribution is to demonstrate that a simple mathematical model, amenable to implementation in an Excel spreadsheet, can accurately predict the evolution of an epidemic event at a local level (i.e., in any major urban area). This model may be extremely valuable for government officials who must predict, with high fidelity, the progression of an epidemic event to better design their action strategies. Moreover, the democratization of the modeling of complex epidemic events will empower citizens, enabling them to forecast, decide, and evaluate. For instance, using this simple model, virtually any citizen could assess, in real time, the efficacy of the actions of her/his society in the face of an outbreak.

## Rationale of the model formulation

Here, we construct a very simple epidemiological model for the propagation of COVID-19 in urban areas.

**Figure 1:**
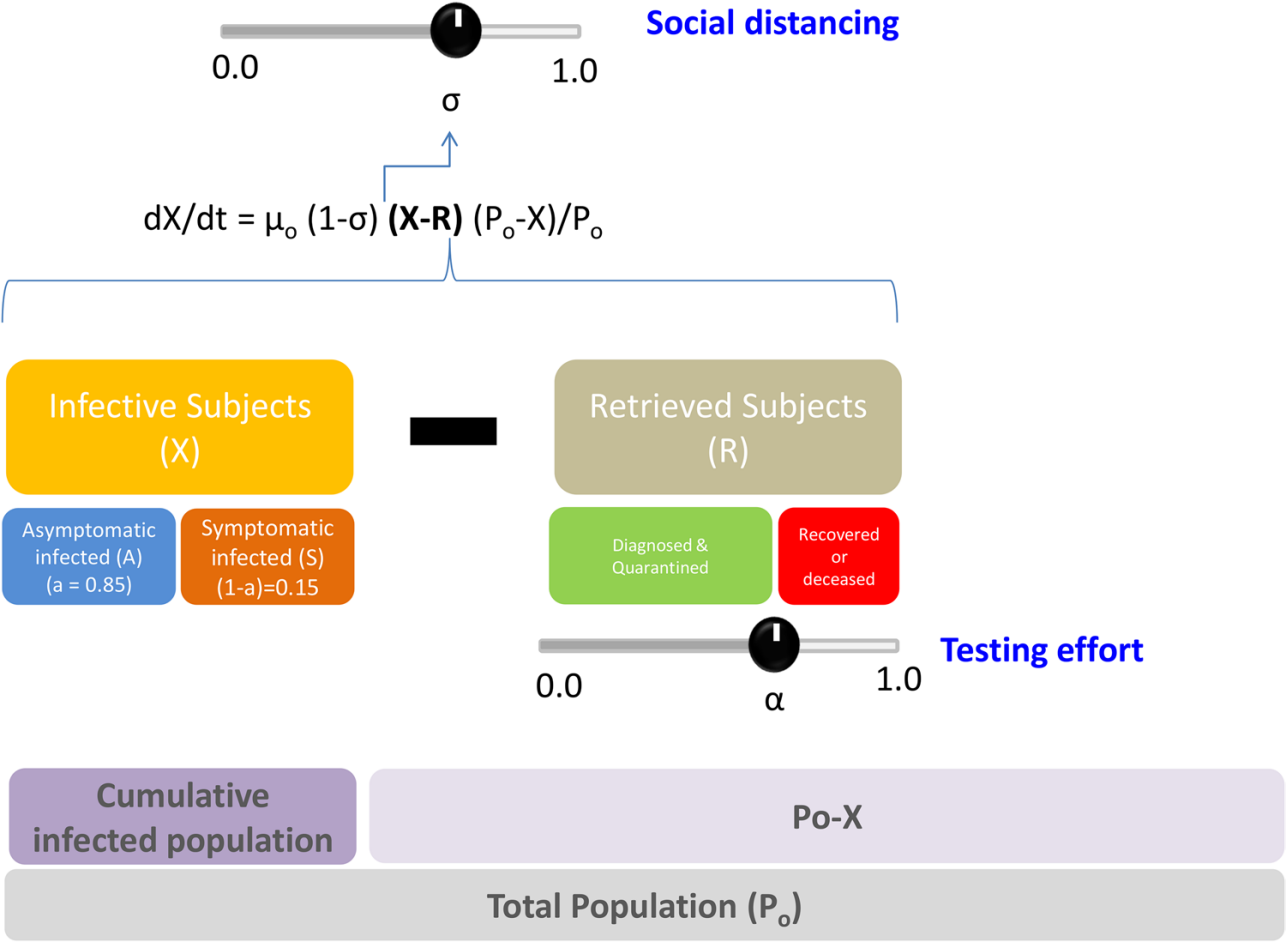
Model formulation. (**A**) Schematic representation of the model. This novel multi-compartment demographic model formulation considers that new infections are proportional to (X–R; infected-retrieved). Demographic elements are directly integrated into the model (P_o_, total population). The positioning and size of different bars indicates relationships between components. For instance, as the cumulative infected population progresses, the susceptible population (P_o_–X; total population minus infected individuals) is reduced. The social distancing (σ) and the testing effort (α) are explicitly stated as the two main parameters that modify the epidemic progression.

The model is based on a set of differential equations and considers two variable populations of individuals: infected (X) and retrieved (R) (Fig. 1). The cumulative number of infected patients (X) is the total number of subjects among the population that have been infected by SARS-CoV-2. The number of retrieved patients should be interpreted as the number of individuals that have been retrieved from the general population and are not contributing to the propagation of COVID-19. Retrieved subjects include subjects who have recovered from the infection and do not shed virus, quarantined individuals, and deceased patients. Importantly, the model assumes that infection results in (at least) short-term immunity upon recovery. This assumption is based on experimental evidence suggesting that rhesus macaques that recovered from SARS-CoV-2 infection could not be reinfected^22^. However, the acquisition of full immunity to reinfection has not been confirmed in humans, although it is well documented for other coronavirus infections, such as SARS and MERS^23,24^.

Two sets of parameters, demographic and clinical/epidemiological, determine the interplay between these two main populations and other subpopulations that include asymptomatic infected (A), symptomatic infected (S), and deceased (D) individuals. Clinical parameters include an intrinsic infection rate constant (µ_o_) that is calculated from the initial stage of the pandemic in that particular region; the fraction of asymptomatic patients (a); the delay between the period of viral shedding by an infected patient (delay_r), the period from the onset of shedding to the result of first diagnosis and quarantine in the fraction of patients effectively diagnosed (delay_q); and the fraction of infected patients effectively diagnosed and retrieved from the population (α). Demographic parameters include the population of the region (P_o_), the extent of social distancing (σ), and the fraction of infected individuals retrieved from the population due to massive and effective testing (α) (Fig. 1). The model is based on a set of two simple differential equations.

1 $$dX/dt \, = \, \mu_{o} (1 - \sigma ) \, \left( {X - R} \right) \, \left( {P_{o} - X} \right)/P_{o} ,$$

2 $$dR/dt \, = \, \alpha \mathop \smallint \limits_{t = 0}^{t = t - delay\_q} dX/dt {+} \, (1 - \alpha )\mathop \smallint \limits_{t = 0}^{t = t - delay\_r} dX/dt.$$

The first equation of the set (Eq. 1) states that the rate of accumulation of infected habitants (symptomatic and asymptomatic) in an urban area (assumed to be a closed system) is proportional to the number of infective subjects (X − R) present in that population at a given point and the fraction of the population susceptible to infection ((P_o_ − X)/P_o_). Note that the number of infective subjects is given by the difference between the accumulated number of infected subjects (X) and the number of retrieved subjects (R). The fraction of the susceptible population decreases over time as more inhabitants in the community get infected. The proportionality constant in Eq. (1) (µ_o_) is an intrinsic rate of infection that is weighted by the effective fractional reduction of social distancing on the population density (1 − σ).

The second equation (Eq. 2) describes the rate at which infected patients are retrieved from the infective population. Eventually, all infected subjects are retrieved from the population of infected individuals, but this occurs at distinct rates. A fraction of infected individuals (α) is effectively retrieved from the general population soon after the onset of symptoms or after a positive diagnosis. Another fraction of infected subjects (1 − α) is not effectively retrieved from the population until they have recovered or died from the disease. Therefore, in our formulation, the overall rate of retrieval (dR/dt) has two distinct contributions, each one associated with different terms on the right-hand side of Eq. (2). The first term accounts for the active rate of retrieving infected patients through the diagnosis and quarantine of subjects testing positive for SARS-CoV-2 infection. For this term, the delay from the onset of virus shedding to positive diagnosis and quarantine (delay_q) is considered short (i.e., about 2 or 5 days), to account for a reasonable time between the positive diagnosis and the action of quarantine. In our model formulation, this term is multiplied by α, the fraction of subjects successfully quarantined after positive diagnostic. A second term relates to the recovery or death of infected patients (symptomatic or asymptomatic) and is represented by the integral of all infected subjects recovered or deceased from the onset of the epidemic episode in the region, considering a delay of 21 days (delay_r), which accounts for the average time of recovery of an infected individual. Note that the simultaneous solution of Eqs. (1) and (2) is sufficient to describe the evolution of the number of asymptomatic individuals (A), symptomatic individuals (S), and deceased patients (D) through the specification of several constants and simple relations.

3 $$a \, dX/dt \, = \, dA/dt,$$

4 $$\left( {1 - a} \right) \, dX/dt \, = \, dS/dt,$$

5 $$m \, \left[ {\left( {1 - a} \right) \, dX/dt \, } \right] \, = dD/dt.$$

Here, **a** is the fraction of asymptomatic subjects among the infected population, (1 − **a**) is the fraction of infected individuals that exhibit symptoms, and **m** is the mortality rate expressed as a fraction of symptomatic individuals.

Please note that in this demographic model (Eqs. 1 and 2; Table 1; Fig. 1), the rate of new infections is corrected by two factors that together define an effective demographic density of the region: (1 − σ) (P_o_ − X)/P_o_. Here (1 − σ) is the current level of activity in the region due to the implementation of social distancing measures (σ). In addition, the factor (P_o_ − X)/P_o_ updates the susceptible population each time step by removing the infected population from the total population.

**Table 1:** Epidemiological data and parameter values used in the model.

Parameter	Definition (units)	Value or [range]	Reference/source
µ_o_	Intrinsic rate of infectivity of COVID-19 (day^−1^) Calculated from confirmed COVID-19 cases during the first stage of the pandemic at Madrid, Spain (Ministy of Health, Spain): (https://www.mscbs.gob.es)	0.36–0.65	Calculated from officially reported data: number of symptomatic COVID-19 positive cases
P_o_	Total population of the city (hab) Provided by model user from actual census data; (i.e.; www.citymayors.com)	[0, 0–15, 500, 800]	From census information
delay_q	Average number of days between infection and positive diagnostics and quarantine (days)	2–5	Assumed based on COVID-19 typical cronology^35^
delay_r	Average number of days between infection and effective recovery or dead (days)	14	^34,36,37^
a	Fraction of asymptomatic subjects among infected. Inferred from serological study conducted in NYC: (www.cnn.com)	0.85	^27^
(1 − a)	Fraction of symptomatic subjects among infected	0.15	
m	Mortality rate expressed as a fraction of symptomatic individuals Continuously updated by Johns Hopkins University (Coronavirus Research Center). https://coronavirus.jhu.edu/	0.069	^42,43^
σ	Social distancing (dimensionless) Parameter provided by model user to simulate a scenario	[0–1]	User defined
(1 − σ)	Fractional reduction in activity in the city due to social distancing	[0–1]	Calculated from σ

The formulation of Eqs. (1) and (2), enables stepwise numerical integration, for example by the Euler method. We have implemented this solution in an Excel spreadsheet (Supplemental File F1). To that aim, differential Eqs. (1) and (2) should be converted into their corresponding equations of differences:

6 $$\Delta {\text{X }} = \, \mu_{{\text{o}}} \left( {{1} - \sigma } \right) \, \left( {{\text{X}} - {\text{R}}} \right) \, \left( {{\text{P}}_{{\text{o}}} - {\text{X}}} \right)/{\text{P}}_{{\text{o}}} \Delta {\text{t,}}$$

7 $$\Delta {\text{R }} = \, \left\{ {\alpha \mathop \smallint \limits_{t = 0}^{t = t - delay\_q} dX/dt {+} \, ({1} - \alpha )\mathop \smallint \limits_{t = 0}^{t = t - delay\_r} dX/dt} \right\}\Delta {\text{t}}{.}$$

For all the simulation results presented here, we set Δt = 1 h = 1/24 day. We have solved this differential set, step by step, updating the values of asymptomatic individuals (A), symptomatic individuals (S), and deceased patients (D), and susceptible population (P_o_ − X) according to Eqs. (3) to (5) (Supplemental File S1).

## Rationale of the election of relevant epidemiological parameters

In the current version of our model, asymptomatic patients are considered part of the population capable of transmitting COVID-19; reported evidence that suggests that asymptomatic subjects (or minimally symptomatic patients) may exhibit similar viral loads^25^ to those of symptomatic patients and may be active transmitters of the disease^5,26,27^. We define the parameter a = 0.85, where **a** is the fraction of asymptomatic within the population. Therefore, (1 − **a**) is the fraction of the population that exhibit symptoms. Our selection of a = 0.85 is based on a recent large-scale serological study conducted in New York City (NYC) to find anti-SARS-CoV-2 antibodies among the population and a computational model^27^. This serological result, which is based exclusively on information from NYC, suggests that ~ 85% of exposed New Yorkers were asymptomatic or exhibited minor symptoms. Based on this (as yet still unpublished) data, we assumed a symptomatic fraction of only 15% in the calculations and forecasts presented here. This assumption should be regarded as speculative, since the information specific for the ratio between symptomatic and asymptomatic COVID-19 patients, although available, is not conclusive at this point^28–30^. These values are also consistent with the high number of asymptomatic infected subjects estimated for other pandemic events. The percentage of asymptomatic infections during pandemic Influenza A/H1N1/2009, based on epidemiology studies founded in serological analysis in a vast range of geographical settings, has been estimated between 65 and 85%^31^; up to 20–40% of the population in urban areas (i.e., Monterrey in México, and Pittsburgh in USA)^32,33^ exhibited specific antibodies against Influenza A/H1N1/2009 regardless of experiencing symptoms, while the fraction of confirmed symptomatic infections was lower than less than 10%.

In addition, the average time of sickness was set at 21 days in our simulations, as this is within the reported range of 14–32 days^34,35^, with a median time to recovery of 21 days^36^. Studies show that high numbers of viral particles (~ 10^5^ viral copies mL^−1^) can be found in saliva from COVID-19 patients even at day 20 after the onset of symptoms^37^. Therefore, we assume that all those infected not quarantined could continue to transmit the virus until full recovery (21 days). Similarly, asymptomatic patients are only removed from the pool of susceptible persons after full virus clearance. The fraction of deceased patients (m) was calculated as m = 0.023 of those infected 14 days before. This mortality percentage (case fatality rate) lies within the range reported in recent literature for COVID-19^14,38–40^. The time lapse of 14 days between the onset of disease and death was statistically estimated by Linton et al. in a recent report^41^.

The straightforward implementation of the model in Excel (Supplemental File S1), using the set of parameters described before, allows the calculation of all populations (X, A, S, and D) every hour. Note that this model enables the description of the progressive exhaustion of the epidemic, as expected by the progressive depletion of the susceptible population. Next, we discuss criteria for selection of the values of µ_o_ based on the initial behavior of the COVID-19 pandemic at different urban areas around the globe.

## Estimation of specific epidemic rate values

We further propose that µ_o_ may be calculated from actual epidemiological data corresponding to the first exponential stage of COVID-19 local epidemics. We determined the appropriate ranges of values for µ_o_ by analyzing publicly available data from different websites that continuously monitor the progression of confirmed cases of COVID-19 for different nations (Table 2).

**Table 2:** Websites displaying COVID-19 data in practically real time.

Our World in data:
https://ourworldindata.org/coronavirus;
Worldometer
https://www.worldometers.info/coronavirus/about/#sources
El País
https://elpais.com/sociedad/2020/03/16/actualidad/1584360628_538486.html
Coronavirus COVID-19 Global Cases by the Center for Systems Science and Engineering (CSSE) at Johns Hopkins University (JHU)
https://gisanddata.maps.arcgis.com/apps/opsdashboard/index.html#/bda7594740fd40299423467b48e9ecf6
Wikipedia, The Free Encyclopedia
https://en.wikipedia.org/wiki/2020_coronavirus_pandemic_in_Iran
https://en.wikipedia.org/wiki/COVID-19_pandemic_in_South_Korea
Fast-trackcities.org
https://www.fast-trackcities.org/content/data-visualization-mexico-city-covid
New York City Government. Health. COVID-19 Data. 2020
https://www1.nyc.gov/site/doh/covid/covid-19-data.page
CONACyT. COVID-19 México. Tablero México. (2020)
https://coronavirus.gob.mx/datos/

**Figure 2:**
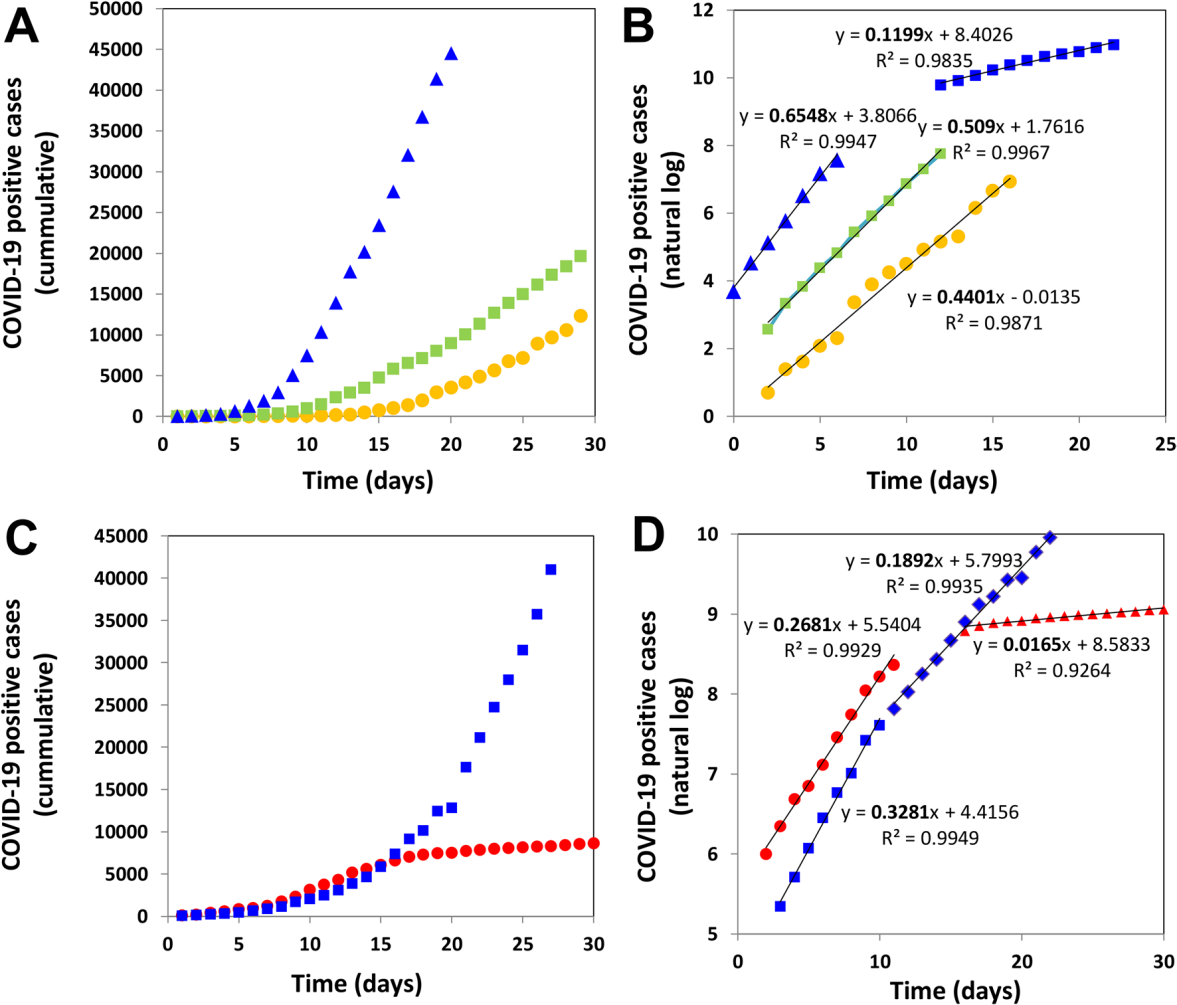
Epidemiological data related to the onset of a COVID-19 pandemic in different regions. (**A**) Cumulative number of positive cases of COVID-19 infection in Spain (yellow circles), Iran (green squares), and NYC (blue triangles) during the first days after the outbreak. (**B**) Natural logarithm of the cumulative number of positive cases of COVID-19 infection in Spain (yellow circles), Iran (green squares), and NYC (blue triangles and squares). (**C**) Cumulative number of positive cases of COVID-19 infection in Italy (blue squares) and South Korea (red circles). (**D**) Natural logarithm of the cumulative number of positive cases of COVID-19 infection in Italy (blue squares and diamonds) and South Korea (red circles and triangles). Two clearly distinctive exponential stages are observed in the case of the NYC and South Korean progression.

Figure 2A shows the progression on the number of COVID-19 positive cases in different regions, namely Spain (mainly Madrid), Iran (mainly Tehran), Italy, and New York City (NYC). We have selected these data sets to illustrate that the evolution of the epidemic has a local flavor that mainly depends on the number of initial infected persons, the demographic density, and the set of containment measures taken by government officials and society. Figure 2B shows the natural log of the cumulative number of infections over time for the same set of countries. This simple plotting strategy is highly useful for analyzing the local rate of progression of the pandemic.

In an initial stage, the local epidemic progression is consistent with a simple first order exponential model d(X)/dt = µ [X], where [X] is the number of initially infected subjects. Then the integral form of this equation renders the linear equation: ln X/X_o_ = µ × t. During the exponential phase, a straight line should be observed, and the slope of that line denotes the specific rate (µ_o_) of the epidemic spreading. Note that COVID-19 has exhibited a wide range of spreading rates in different countries (from ~ 0.12 to ~ 0.65 day^−1^). Note also that µ is related to the doubling time (t_d_), often reported in population and epidemiological studies, by the equation t_d_ = Ln 2/µ. Therefore, ranges of doubling times between 1.07 and 5.77 days are observed just among these three regional cases. T_d_, which can also be defined as a function of time t_d_(t), gives a reliable measure of the efficiency of the containment policy^44,45^.

Different exponential stages, perfectly distinguishable by their exhibition of different slopes (Table 3), may be observed within the same time series. For instance, the outbreak in NYC (Fig. 2B; blue symbols) was first described by an extremely high slope (µ_o_ = 0.654 day^−1^). However, after a series of measures adopted in NYC by the federal, state, and local governments, the specific growth rate of the epidemics fell to µ = 0.119 day^−1^.

**Table 3:** Specific infection rates (µ_o_) and associated doubling times (t_d_) for COVID-19 infection in different geographic regions.

Territory	Temporality	µ (day^−1^)	t_d_ (day)
Spain (Madrid)	Initial	0.440	1.575
Italy	Initial	0.328	2.113
Italy	After stringent measures	0.189	3.667
Iran	Initial	0.491	1.412
Iran (Tehran)	Initial	0.509	1.361
Germany	Initial	0.280	2.475
NYC	Initial	0.655	1.058
NYC	After measures	0.120	5.776
South Korea	Initial	0.268	2.586
South Korea	After stringent measures; massive testing	0.016	43.322
France	Initial	0.379	1.828
France	After measures	0.161	4.311
Mexico	Initial	0.330	2.100

The last point is extremely important, since two drastically different slopes can be observed before and after a package of adequate measures within the same territory. In addition, two localities that experienced similar initial specific epidemic rates may exhibit dramatically different evolutions as a function of the initial response of government and society (Fig. 2C,D). For instance, while the COVID-19 epidemics in Italy and South Korea exhibited similar µ_o_ values (0.328 and 0.268, respectively), the Italian outbreak decreased the growth rate to 0.189 after emergency measures, while South Korea set an example by effectively and rapidly lowering the specific epidemic rate to nearly 0 in just 2 weeks.

## Validation and predictions: effect of social distancing and testing

Overall, the model is capable of closely reproducing the progression of reported cases for urban areas. We found that, adapting the model to a particular locality is straightforward and only requires (a) the declaration of the population of the urban area, and (b) the selection of a t_d_ value (time to doubling the name of infections) or µ_o_ (initial infective rate); (ln 2 = µ_o_ t_d_). Note that our model is formulated in terms of values of the specific epidemic growth rate (µ_o_ for the onset of the epidemic and µ for later times). However, expressing the specific epidemic rate in terms of doubling time (t_d_ = Ln 2/µ) may be more practical and simpler to communicate and understand (Table 3). The selection of µ_o_ (t_d_) can be easily done by fitting the prediction to the initial set of reported cases of infection. In our experience, four to five reliable data points are needed for a good fit.

**Figure 3:**
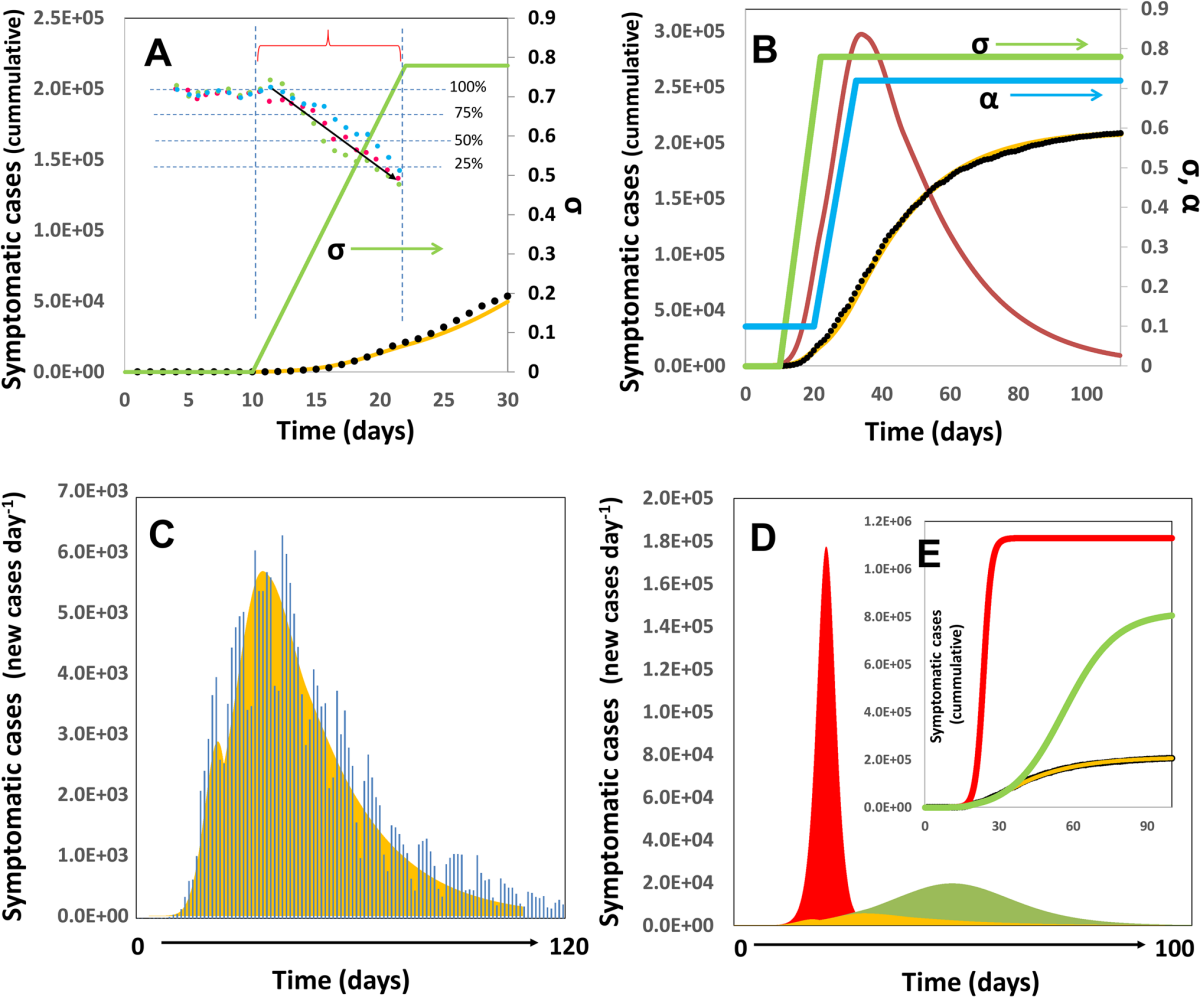
Progression of the COVID-19 Pandemic in NYC. (**A**) Initial evolution of the number of positive cases of COVID-19 in NYC. Actual data points, as officially reported, are shown using black circles. Simulation predictions are described by the yellow line. The profile of social distancing values used in simulations (σ) is shown as a green line. Relative change in visits to different type of places in NYC (modified from Ref.^46^) as reported by Bakker et al. (modified from Ref.^46^): food (green circles), shopping (red circules), and city/outdoors (blue circles) (**B**) Model prediction of the total number of symptomatic patients through the months of March and May. Actual data points, as officially reported, are shown using black circles. Simulation predictions are described by the yellow line. The profiles of social distancing (σ) and testing effort (α) are shown as green and blue lines, respectively. The value of (X–R), determinant of the progression of the infection among population, is shown as a red line. (**C**) Model prediction (yellow) and actual number of new cases of COVID-19 per day (as reported by the NYC authorities; blue bars; https://www1.nyc.gov/site/doh/covid/covid-19-data.page) during the period from March 1 to June 30, 2020. (**D**) Prediction of the number of new cases of COVID-19 per day if no containment actions were adopted (red area), if only social distancing were adopted (in accordance with the green profile of σ values in A and B) (green area), or in the actual case were social distancing combined with intensified testing and quarantine were adopted (yellow area). The inset show the cumulative number of cases predicted by the model for the same scenarios previously described. Actual data points corresponding to the officially reported number of cumulative COVID-19 cases in NYC are shown as black dots.

We have run different scenarios to validate the predictive capabilities of our epidemic model for COVID-19. First, we illustrate the use of the model by recreating the pandemic progression in NYC, one of the most densely urban areas worldwide. Figure 3 shows the predicted trend of the pandemic in NYC during the initial stage of the pandemic wave from March to May, 2020. We set (P_o_ = 8,350,000) and selected a value of µ_o_ = 0.655 (t_d_ = 1.058) for the first week of this simulation. By the second week of March, stringent measures of social distancing were imposed in NYC^46^. Social distancing has been regarded as the one of the most effective buffering measures for local COVID-19 epidemics^8,47,48^. The evaluation of social distancing was straightforward. In the demographic model, we have defined σ as a dimensionless social distancing parameter, while 1 − σ is the remaining fraction of activity in a society after the application of social distancing measures that reduce the level of activity in an σ fraction. Accordingly, in the Excel implementation of the model, we can multiply the value of µ (the specific infection rate) by a factor of (1 − σ) to obtain a proper fit for the new trend on actual cases and to calculate the impact of distancing measures that would diminish social contact. For example, a constant value of σ = 0.25 means that social activities will be decreased by 25%. Similarly, we multiplied µ by (1 − σ) = 0.50 to simulate the effect of a scenario of social distancing that would diminish close social interaction by 50% (see Supplementary Fig. S1). In practice, social distancing must be a function of time. Indeed, measures aimed to enforce social distancing are normally applied progressively. In the Excel implementation of the demographic model, we have reserved a column to provide values for σ. In this way, the user can define σ as a constant or as a function of time, namely σ(f). We evaluated the effect of different degrees of social distancing on the shape of the epidemic curve for NYC to identify plausible ranges of σ to use in the NYC simulations (Supplementary Fig. S1)^46^. Social distancing has a clear buffering effect on the epidemics, delaying the occurrence of the peak of infections and distributing the number of cases across a longer time span. This is remarkably important as it provides time for proper attention to patients with severe symptomatology^9^. The effect of anticipating measures of social distancing has a moderate effect on retarding the infection curve but not on decreasing the cumulative number of infections (Supplementary Fig. S1). This moderate gain of time provides additional leeway for planning interventions or allocating resources, with time being gold during pandemic events. For instance, our results suggest that, for an urban area such as NYC, imposing measures that guarantee a social distance (α = 0.5) equivalent to a decrease in demographic density of 50% will delay the peak of maximum number of infections by 15 days (from day 23 to day 38) and will decrease its intensity from ~ 175,500 to ~ 80,600 new cases of infection per day. In turn, this implies a lower demand for hospital beds per day during the epidemics and may mark the difference between a manageable crisis and a public health catastrophe^9, 47^.

We conducted a series of simulations by varying the values of α = 0.5 to fit the actual data of cumulative number of reported cases of COVID-19 and the number of new cases per day. The results of our simulations suggest that strict measures of social distancing had to be rapidly implemented in NYC during the first weeks of the pandemic episode and that the measures of social distancing imposed in NYC were equivalent to a decrease in the effective demographic density of more than 70% (σ > 0.70) in a few days. The comparison between the actual and the predicted scenarios in terms of new cases is presented in Fig. 3A. In these simulations, we set a linear ramp of values of effective social distancing from σ = 0.0 to 0.75 in twelve days, which is consistent with reports on the decrease in mobility in NYC between March 10 and March 23, 2020^46^.

The analysis presented in Fig. 3A for NYC only considers the effect of social distancing. This simple embodiment of the model may enable an accurate forecast of pandemic scenarios in territories (or time periods) in which massive testing campaigns were not enforced (e.g., Mexico City; a case that we will analyze later). However, the long-term analysis of the progression of COVID-19 in NYC required the consideration of testing campaigns. Our demographic model allows a definition of the fraction of infected subjects (σ), and the span of days between infection and effective quarantine, given a positive diagnostic (delay_q). We explored different values of α for a fixed assumed value of delay_q (i.e., delay_q = 4 days) and found a set (progression) of α that reasonably reproduces the progression of the first wave of COVID-19 in NYC during the first wave of the pandemic episode. Figure 3B shows the number of cumulative cases predicted and reported in NYC (from March to May 2020) and the profile of values of social distancing (σ) and testing intensity (α) used to generate the predicted profiles. Our simulation results (Fig. 3B,C) suggest that an intensive testing campaign had to be enforced to contain the pandemic wave, and we were able to reproduce the actual progression of pandemic COVID-19 in NYC by setting a linear ramp of α values form 0 to 0.76 in just two weeks, from March 20 to April 7, 2020. This is somewhat consistent with the information now available on the number of PCR tests conducted in the USA during March and April 2020. In general, the USA is one of the leading countries in terms of the number of PCR tests performed during the first semester of 2020, and NYC was the first epicenter of COVID-19 in America^49,50^. PCR-based testing in the USA started in mid-March (i.e., mainly NYC) and increased rapidly to more than 100,000 PCR tests daily. The relevance of wide-scale testing to control the progression of COVID-19 in urban areas has been discussed widely in literature. In agreement, the results of our simulations suggest that massive testing, combined with a social distancing (σ ~ 0.75), were key to facing the COVID crisis in NYC. Figure 3D shows the predictions of the number of daily cases of COVID-19 in NYC in different scenarios (i.e., with no intervention, with only social distancing [σ ~ 0.75; α = 0.10], and with social distancing and aggressive testing as actually implemented). Note that in the context of our work, no intervention implies that persons diagnosed as positive for COVID-19 are still quarantined (α = 0.10). Our simulations predict that the total number of cases positive for COVID-19 would have exceeded 1.3 million in the absence of social distancing measures during the first 100 days of the epidemic. The implementation of social distancing alone would have resulted in nearly 800,000 positive cases within the same timeframe. The combination of social distancing and aggressive testing decreased this sum to nearly 200,000 and avoided a human catastrophe in one of the most densely populated cities in the world.

**Figure 4:**
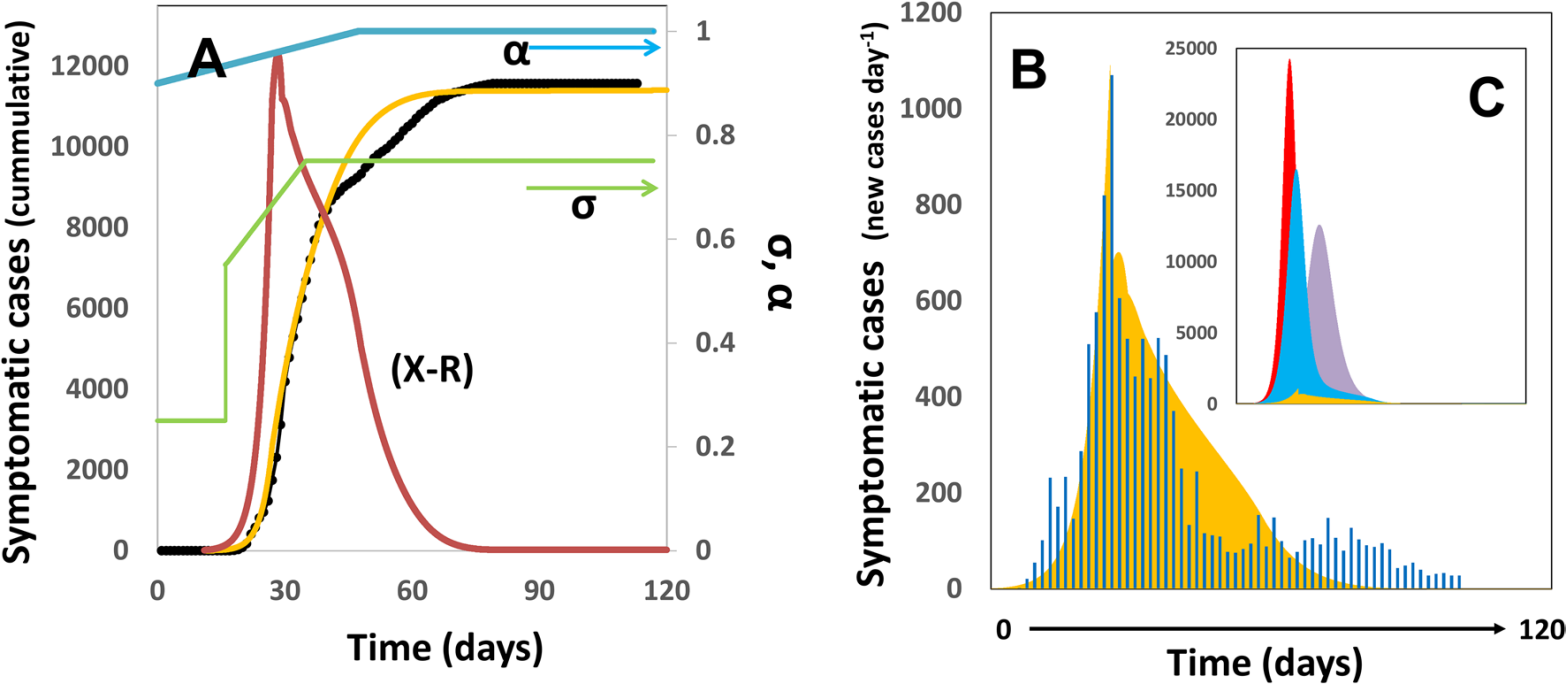
Progression of the COVID-19 Pandemic in South Korea. (**A**) Model prediction of the total number of symptomatic patients through the months of February and May. Actual data points, as officially reported, are shown using black circles. Simulation predictions are described by the yellow line. The profiles of social distancing (σ) and testing effort (α) are shown as green and blue lines, respectively. The value of (X–R), determinant of the progression of the infection among population, is shown as a red line. (**B**) Model prediction (yellow) and actual number of new cases of COVID-19 per day (blue bars; https://en.wikipedia.org/wiki/COVID-19_pandemic_in_South_Korea) during the period from February to May, 2020. (**C**) Prediction of the number of new cases of COVID-19 per day if no containment actions were adopted (red area); if only intensified testing and quarantine were adopted [in accordance with the blue profile of α values in (**A**)] (blue area); if only social distancing were adopted [in accordance with the green profile of σ values in (**A**)] (purple area); or in the actual case were social distancing combined with intensified testing and quarantine were adopted (yellow area).

We also explored the adequacy of our demographic model for describing the dynamics of the first pandemic wave in South Korea. South Korea based its strategy of COVID-19 control on widespread testing, efficient contact tracing, and self-quarantine programs for suspected positive individuals^51^. South Korea implemented an open public testing program early in February and made it available even to asymptomatic people^49,50^. Testing quickly ramped up to more than 10,000 tests per day, mainly in the city of Daegu (with a metropolitan area of nearly 2.5 million people). Modeling the pandemic evolution in South Korea was more challenging than that in NYC. However, we were able to closely reproduce the dynamics of the first wave of pandemic COVID by setting an aggressive slope of social distancing (i.e., self-quarantine, use of masks, avoidance of public gatherings) as well as an aggressive testing campaign (α ~ 0.98). This means that, to properly fit the actual data on cumulative cases and new infections per day (Fig. 4A,B), we had to assume that the testing effort in South Korea resulted in finding and effectively quarantining nearly 100% of all infected persons within a few days (i.e., within 2 days in our simulations). Our model suggests that the early adoption of wide spread testing and contact tracing to quickly finding infected individuals, in combination with social distancing, is much more effective than only social distancing or massive testing alone (Fig. 4C).

## Prediction in real time: pandemic progression in Mexico City

**Figure 5:**
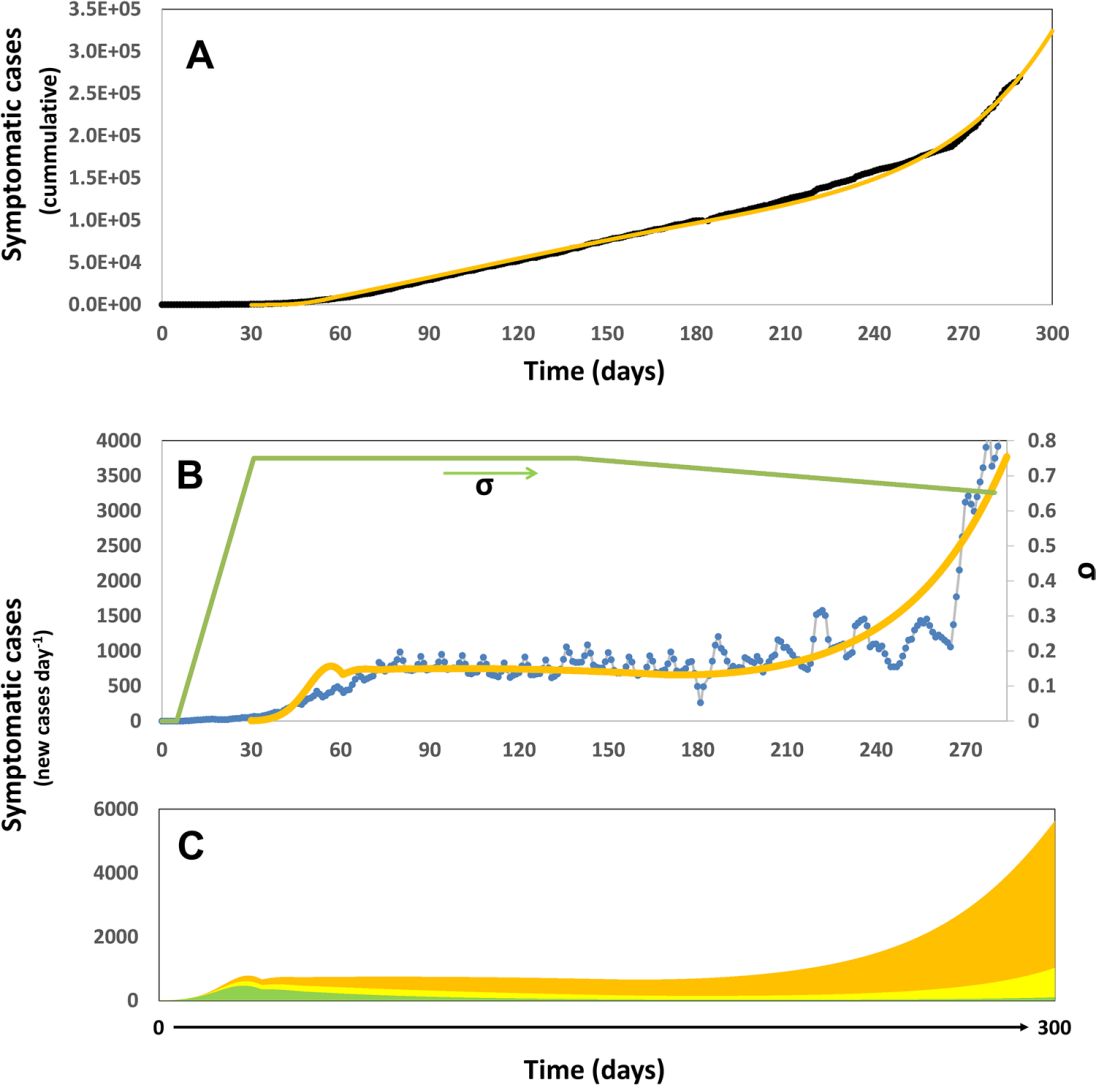
Progression of the COVID-19 Pandemic in Mexico City. (**A**) Model prediction of the total number of symptomatic patients through the months of Mach and December, 2020. Actual data points, as officially reported, are shown using black circles. Simulation predictions are described by the yellow line. The profile of social distancing (σ) is shown as a green line. A constant value of α = 0.10 was used in this simulation. (**B**) Model prediction (yellow line) and actual number of new cases of COVID-19 per day (as reported by the Mexican authorities; blue line; https://www.fast-trackcities.org/content/data-visualization-mexico-city-covid) during the period from February to December, 2020. (**C**) Prediction of the number of new cases of COVID-19 per day if the testing effort would have been doubled (light yellow area) or tripled (green area). The simulation of the actual pandemic scenario is also shown (yellow-orange area).

We also have followed the onset and progression of the COVID-19 pandemic in México City, the most industrialized and most populated city in México. We set (µ_o_ = 0.33; t_d_ = 2.1) based on proper fitting to the first set of the official values of COVID-19 infection announced for México City by the local authorities from March 6 to March 18, 2020 (https://www.fast-trackcities.org/content/data-visualization-mexico-city-covid). Since then, the simulation results have closely predicted the actual values for more than 300 days, as officially reported from March 19 to December 20 (Fig. 5A,B). The COVID-19 evolution in Mexico City exhibits remarkable differences with respect to those observed in other countries. For instance, the first pandemic wave has not yet ended (Fig. 5A,B) at the time of this writing. An epidemic peak was observed in May 2020. After the peak, the number of new cases per day remained nearly constant for months. The number of daily cases has increased from October to December 2020 and has now reached alarming values at the end of 2020 (i.e., more than 5000 cases per day). The Mexican strategy to face COVID-19 has been guided by the enforcement of social distancing since the onset of the epidemics (i.e., March 10, 2020). A system of four colors (i.e., red, orange, yellow, and green) was established by the government officials to allow continuous communication of the status of the pandemic in the different regions across Mexico. In this scale of colors, red conveys the maximum level of alert. Colors are also associated with the economic and recreational activities that are allowed and the level of social distancing enforced. Mexico City went from red to orange in June 2020 and back to red in December 2020. Then, the level of enforced social distancing could be considered as high (arguably above 50%) during the pandemic progression. However, wide scale testing has not been considered as part of the official strategy to face COVID-19, and diagnostics have only been done upon request and mainly for symptomatic individuals. Indeed, Mexico has been regarded as one of the countries that have conducted a low number of tests. At the time of this writing, Mexico has conducted 23 tests per 1000 inhabitants. By contrast, as of December 2020, the USA and South Korea had conducted 688 and 71.65 tests per 1000 inhabitants (https://ourworldindata.org/coronavirus)^50^. In December, México, the USA, and South Korea, were performing 0.10, 3.96, and 0.839 tests per 1000 inhabitants daily, respectively^49,50^. Consistent with these data, our demographic model nearly reproduced the entire progression of pandemic COVID-19 in Mexico City by considering a basal level of testing (α = 0.10) and a set of values for social distancing larger than 60% (σ > 0.60). Figure 5A shows the agreement between the actual and simulated cumulative numbers of COVID-19 cases and the profile of values for social distancing used to produce a good fit. Figure 5B shows a comparison between the actual and predicted numbers of daily new cases of COVID-19 in Mexico City. Our analysis suggests that the sudden increase in the slope of the number of daily new cases that has been observed by the end of 2020 was originated by a progressive relaxation of the social distancing (i.e., a linear change in the α values form 0.75 to 0.68 during 150 days).

Our simulations also suggest that the effect of testing intensification could have been key to extinguishing the pandemic wave in the case of Mexico City. Figure 5C shows the predicted effect of doubling (α = 0.20; yellow shaded area) and tripling (α = 0.30; green shaded area) the testing intensity. Based on this demographic model, the cumulative number of COVID-19 cases in Mexico´s capital could have been reduced from ~ 270,000 to ~ 75,300 (a reduction of 72%) by intensifying the testing effort twofold (i.e., ~ 50 tests per 1000 inhabitants).

## Concluding remarks

Scenarios such as those unfolded in Iran, Italy, NYC, Mexico City, England or Spain emphasize the importance of forecasting for planning ahead during epidemic events.

This contribution shows the prediction potential of an extremely simple simulation tool that can be used by practically any citizen with basic training in Excel. We used a set of differential equations, recent epidemiological data regarding the evolution of COVID-19 infection, and basic information on the characteristics of COVID-19 infection (i.e., time from infection to recovery, case mortality rate) to accurately recreate or predict the progression of the COVID-19 in three urban areas with different demographic characteristics (i.e., NYC in USA, Daegu in South Korea, and Mexico City in México). We showed that the model can be adapted to closely follow the evolution of COVID-19 in densely populated urban areas by simply adjusting parameters related to demographic characteristics (i.e., total population) and aggressiveness of the response from a society/government to epidemics (i.e., social distancing and testing intensity).

One important attribute of this model is that it is amenable to implementation in Excel. This greatly facilitates its widespread use. We anticipate that policy- and decision-makers, scientists, graduate students, and regular citizens (with only a basic training in Excel) will be able to use this model. In addition to being user friendly, the model is also very flexible and enables the simulation of a wide variety of scenarios (i.e., COVID progression under different degrees of social distancing and testing effort) and enables rational planning (i.e., prediction of hospital bed occupancy, design of testing campaigns, and reinforcement/redirection of social distancing strategies). Simple modifications will enable the use of this model for the evaluation of the effect of different vaccination strategies. Finally, the model can be easily adapted to epidemic events related to any other viral or bacterial pathogen by inputting the corresponding epidemiological parameters.

## Supplementary Information

Supplementary Information 1.

Supplementary Information 2.
