# Supplementary material for: Modeling COVID-19 epidemics in an Excel spreadsheet to enable first-hand accurate predictions of the pandemic evolution in urban areas
Source: Sci Rep. 2021 Feb 22;11:4327. doi: 10.1038/s41598-021-83697-w (PMC7900250; doi:10.1038/s41598-021-83697-w)
Supplement: Supplementary file 2 — Supplementary Information 2. [file 41598_2021_83697_MOESM2_ESM.docx]

**Supplementary Figures**

**Modeling COVID-19 epidemics in an Excel spreadsheet to enable first-hand accurate predictions of the pandemic evolution in urban areas**

Mario Moisés Alvarez^1,2,^*, Everardo González-González^1,2^, and Grissel Trujillo-de Santiago^1,3^

^1^ Centro de Biotecnología-FEMSA, Tecnologico de Monterrey, Monterrey 64849, NL, México

^2^ Departamento de Bioingeniería, Escuela de Ingeniería y Ciencias, Tecnologico de Monterrey, Monterrey 64849, NL, México

^3^ Departamento de Ingeniería Mecatrónica y Eléctrica, Escuela de Ingeniería y Ciencias, Tecnologico de Monterrey, Monterrey 64849, NL, México

(*) corresponding author: [*mario.alvarez@tec.mx*](mailto:mario.alvarez@tec.mx)


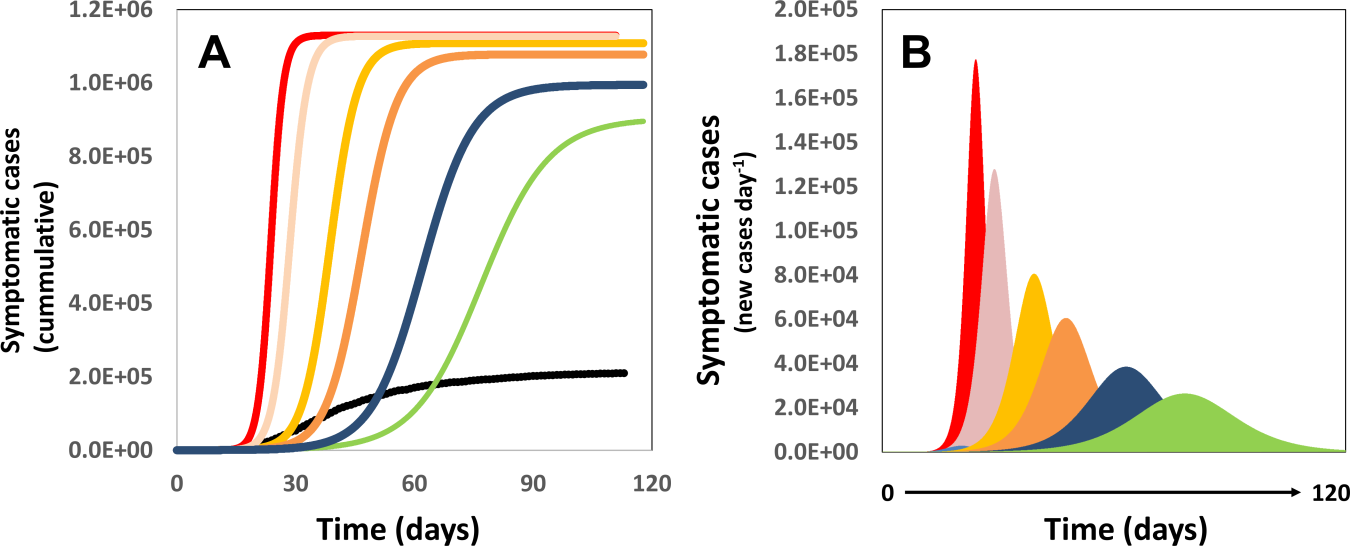


**Figure S1.** **Prediction of the effect of social distancing on the progression of the COVID-19 pandemics in New York City (NYC).** (A) Model prediction of the total number of symptomatic patients from March 1 to May 31, 2020 for different scenarios of social distancing: no social distancing (red line); social distancing effective on March 10, whereby the effective demographic density in NYC is reduced by 25% (pink line), by 50% (yellow line); by 60% (orange line); by 70% (dark blue line), and by 75% (green line). The actual data, as reported by NYC authorities is also presented (black dots). (B) Model prediction for the number of new infections per day for each of the scenarios of social distancing described before.
